# Long-term control for > 9 years with megestrol acetate-letrozole: metastatic endometrioid carcinoma case report

**DOI:** 10.3389/fonc.2026.1729721

**Published:** 2026-02-24

**Authors:** Lixia Liu, Lina Ma, Lu Yang, Wenguang He

**Affiliations:** 1 Shanxi Province Cancer Hospital/Shanxi Hospital Affiliated to Cancer Hospital, Chinese Academy of Medical Sciences/Cancer Hospital Affiliated to Shanxi Medical University, Shanxi, Taiyuan, China; 2 The Genetic Analysis Department, YuceBio Technology Co., Ltd., Shenzhen, China

**Keywords:** endometrioid carcinoma, hr-high expression, letrozole, megestrol acetate, synergistic antitumor effects

## Abstract

**Background:**

Hormone receptor–positive (HR+) endometrial carcinoma (EC) responds to endocrine therapy, but clinical benefit duration is often limited, especially in patients with widely metastatic or recurrent disease.

**Case:**

A 54-year-old woman with HR-high (ER/PR 90%) metastatic EC (FIGO IIIC2) discontinued adjuvant chemotherapy due to severe toxicity. She initiated megestrol acetate and letrozole in March 2017, achieving stable disease (SD) for 38 months. Self-discontinuation (January–July 2020) led to disease progression (new retroperitoneal lymph nodes), but resuming full-dose therapy restored SD for 49 months. After dose reduction (July 2023), the disease progressed again at 12 months; re-escalation to full dose induced regression. At last follow-up (OS: 108 months), she maintained SD with minimal toxicity.

**Conclusion:**

This is the first case demonstrating that full-dose megestrol acetate plus letrozole may induce durable antitumor activity in HR-high expression metastatic EC, warranting prospective validation.

## Introduction

Endometrial carcinoma(EC) is the most common gynecologic malignancy in the United States, ranking top five in incidence and mortality among female cancers, and is the fourth most common malignant tumor in women (1). According to GLOBOCAN 2023 data, the global incidence of EC has shown an upward trend, with regional stability in some areas, and an alarming shift toward younger age groups (2). Thus, exploring strategies to improve survival in patients with advanced or recurrent endometrial carcinoma (EC) is an urgent clinical need.

Approximately 70%–90% of EC patients are Estrogen Receptor (ER)- and/or Progesterone Receptor (PR)-positive, and Hormone Receptor (HR) expression is strongly correlated with responsiveness to endocrine therapy. Based on this, the National Comprehensive Cancer Network (NCCN) (Version 3.2025) recommends endocrine therapies as first-line systemic treatment for recurrent/metastatic HR+ EC (3). However, extending the durable response to endocrine therapy remains a critical clinical challenge, as resistance inevitably develops in most patients, yielding only modest survival benefits with a median overall survival (OS) of 10–15 months (4).

Based on this, we focus on an EC patient who maintained long-term disease control for > 9 years with megestrol acetate plus letrozole, which may provide a potential novel combination of endocrine treatment and drug holiday strategy for HR-high metastatic EC in the era of precision medicine.

## Case presentation

**Figure 1:**
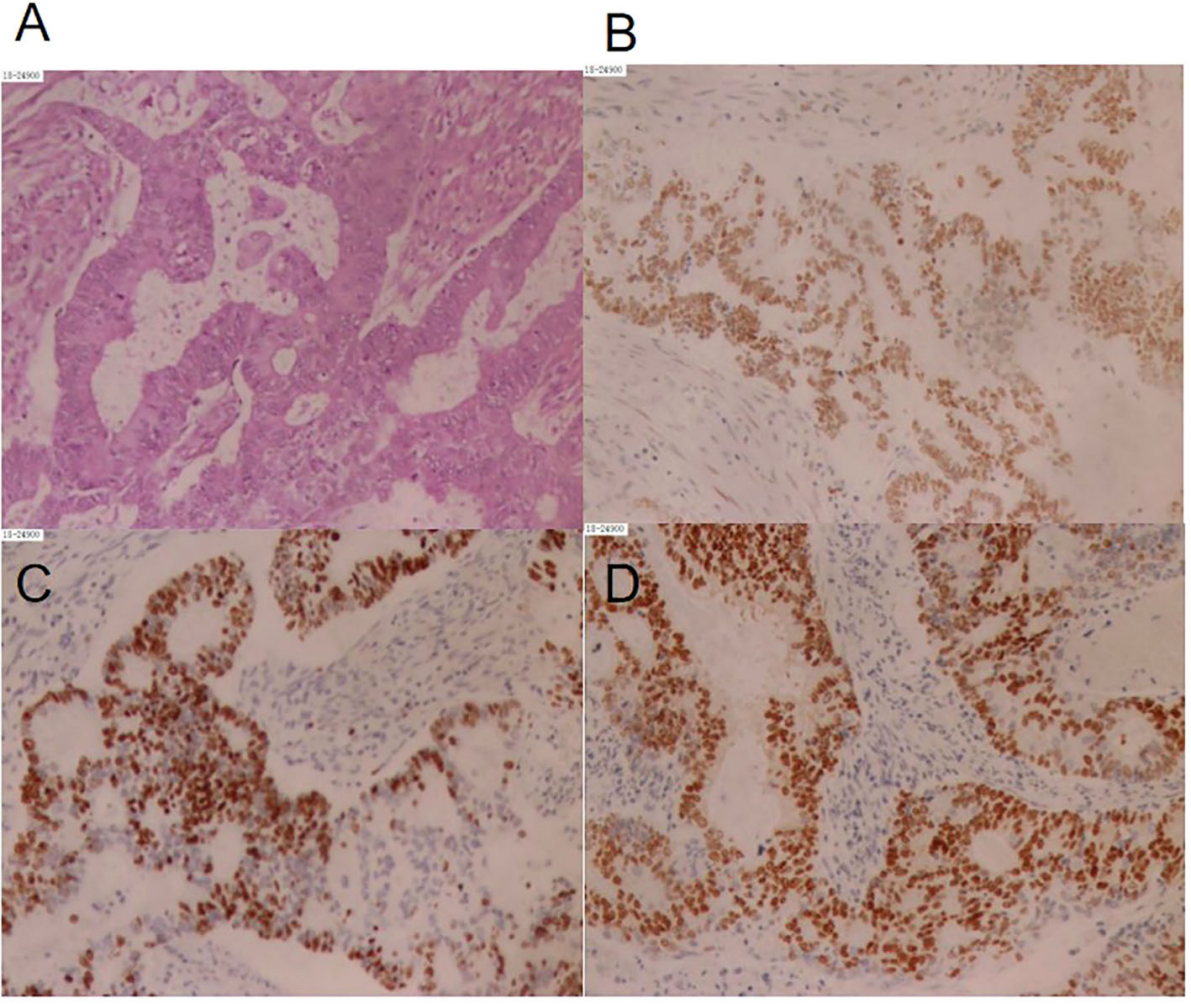
Immunohistochemistry. **(A)** Histology of the tumor tissue, hematoxylin and eosin, original magnification x200; **(B)** immunohistochemistry of ER, original magnification x200; **(C)** immunohistochemistry of Ki67, original magnification x200; **(D)** immunohistochemistry of PR, original magnification x200.

A 54-year-old postmenopausal woman presented to our hospital in June 2016 with 3 months of irregular vaginal bleeding (no history of diabetes, hypertension, or family history of cancer). On July 4, 2016, she underwent an open total hysterectomy, bilateral adnexectomy, and pelvic lymphadenectomy. Postoperative pathology confirmed FIGO stage IIIC2 moderately differentiated EC with multiple lymph node metastases; immunohistochemistry (IHC) showed ER 90%, PR 90%, HER2 1+, and Ki-67 60% (hot spots). (**Figure 1**).

From July 26 to August 22, 2016, she received two cycles of adjuvant chemotherapy (paclitaxel 540 mg IV + nedaplatin 180 mg IV). Still, she discontinued due to severe grade 3 adverse events (AEs) (acid reflux, epigastric pain, generalized pruritus) and grade 2 lower limb weakness (CTCAE 5.0). She then received pelvic radiotherapy (48 Gy in 24 fractions) from October 25 to November 28, 2016, to reduce local recurrence risk.

**Figure 2:**
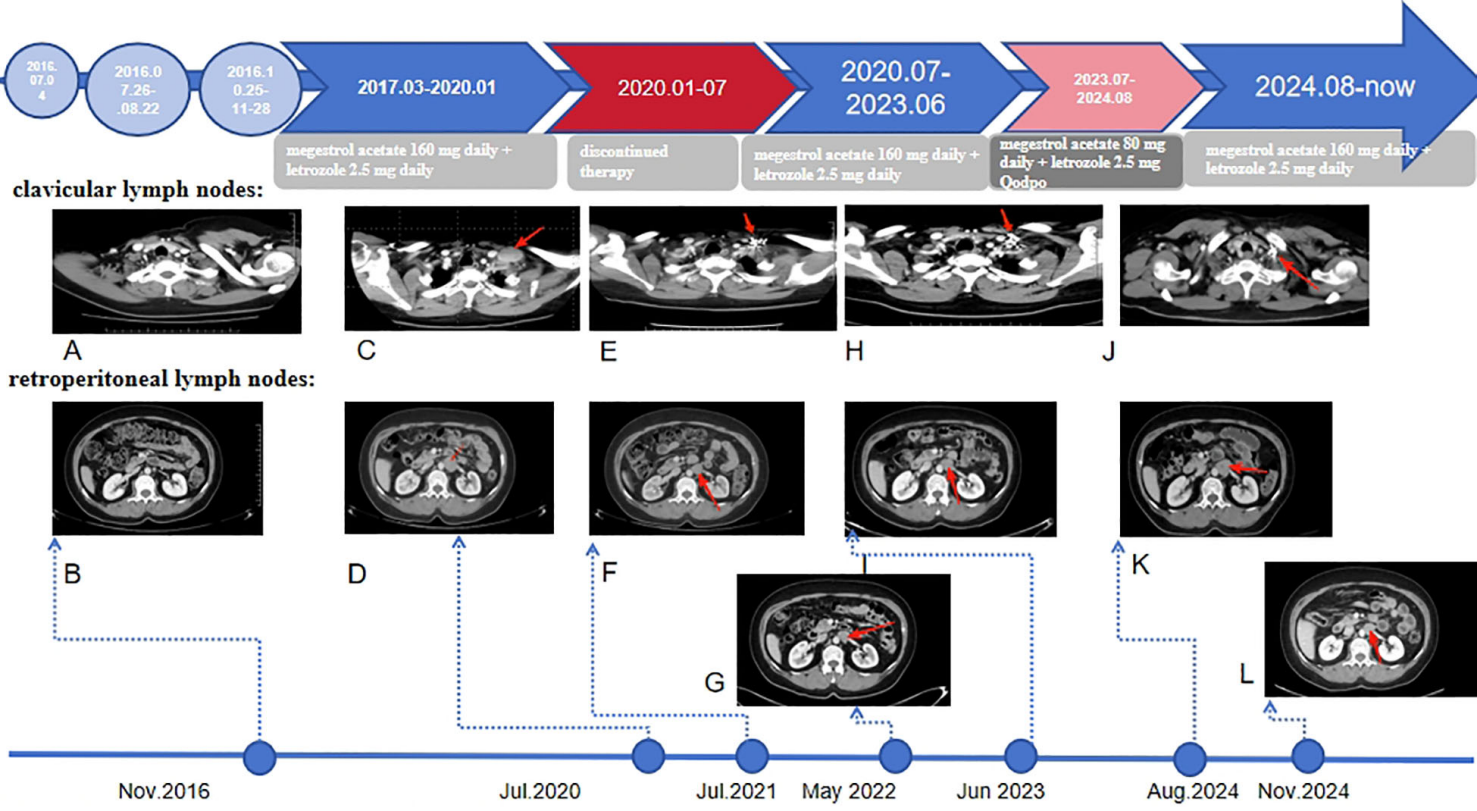
Disease course with treatment history and imaging findings. The red arrows in the image represent changes in clavicular and retroperitoneal lymph nodes. **(A, C, E, H, J)** represent changes in clavicular lymph nodes at different time points. **(B, D, F, G, I, K, L)** represent changes in retroperitoneal lymph nodes at different time points.

**Figure 3:**
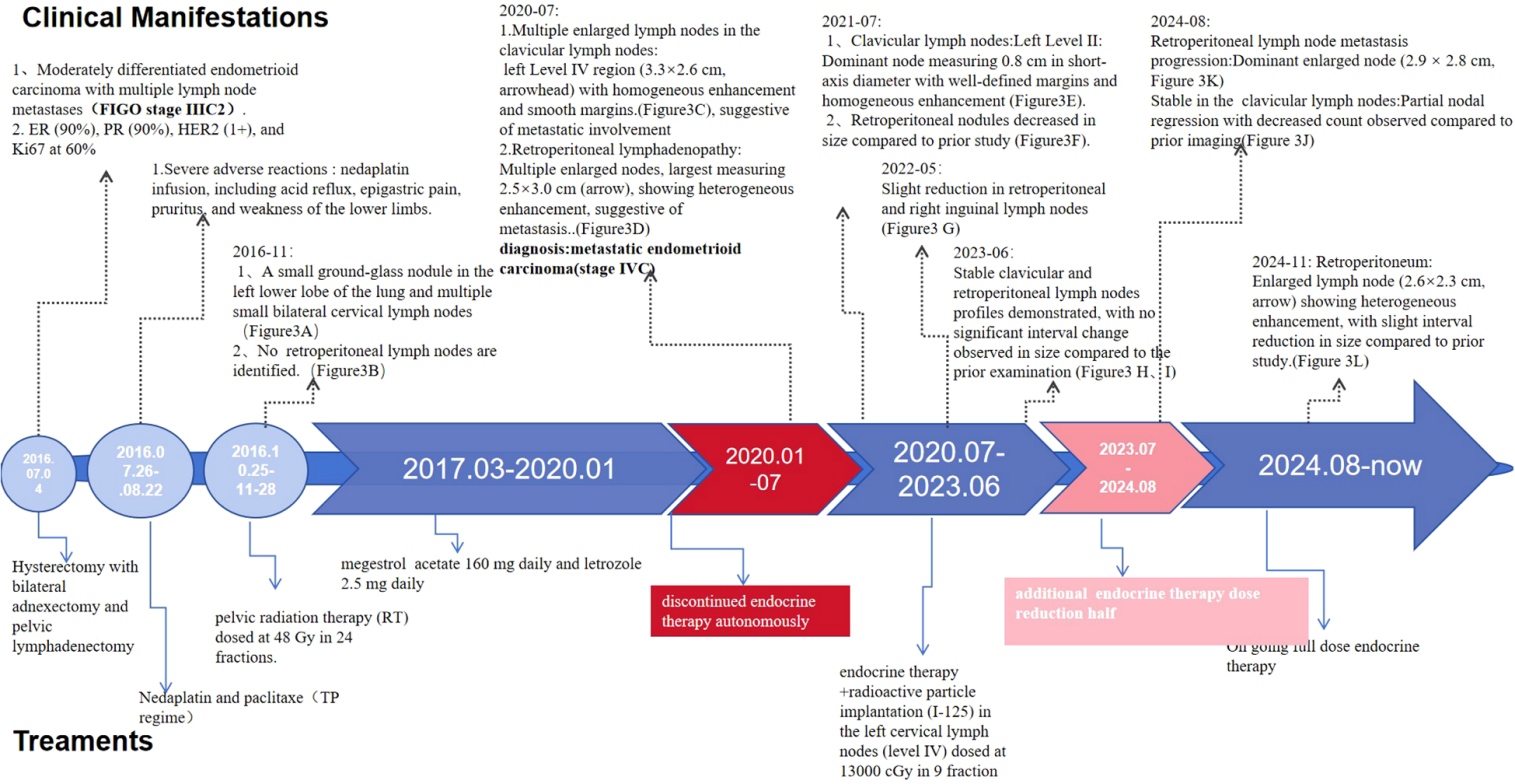
Timeline of key clinical management strategies and manifestations for the patient.

Contrast-enhanced Computed Tomography (CT) in November 2016 showed a 5-mm ground-glass nodule in the left lower lung and bilateral cervical lymph nodes (short-axis <1 cm), with no retroperitoneal lymph nodes (RPLNs). (**Figures 2A, B**). Given chemotherapy intolerance and strong HR positivity, she initiated megestrol acetate (160 mg/d) plus letrozole (2.5 mg/d) in March 2017. Key clinical management strategies and manifestations during treatment and follow-up are detailed in **Figure 3**.

## Long-term follow-up and treatment response

### March 2017–May 2020 (stable disease: progression-free survival = 38 months)

Serial CT scans (May 2017–June 2019) showed SD (per RECIST 1.1), with ECOG PS 0, and only mild grade 1 fatigue (no dose adjustment needed).

### May 2020–July 2020 (PD)

In January 2020, she voluntarily discontinued therapy due to the Coronavirus Disease 2019(COVID-19). On 2020-5-17, CT revealed multiple enlarged lymph nodes in the left cervical level IV region, with morphological features highly suspicious for metastatic involvement. In July 2020, CT imaging identified metastatic lesions in the left cervical lymph node (3.3 × 2.6 cm with homogeneous enhancement) and retroperitoneal lymph nodes (2.5 × 3.0 cm with heterogeneous enhancement). Based on these findings, she was diagnosed with stage IVB recurrence endometrioid carcinoma (FIGO 2009) involving cervical and retroperitoneal lymph nodes. (**Figures 2C, D**).

### July 2020–August 2024 (SD PFS2 = 49 months)

She resumed full-dose endocrine therapy in July 2020 and underwent CT-guided I-125 radioactive seed implantation (13000.0 cGy) for left cervical level IV lymph nodes. Follow-up CT (July 2021–June 2023) showed SD: reduced cervical/retroperitoneal lymph nodes (July 2021: **Figure 2E, F**), further shrinkage of retroperitoneal/inguinal nodes (May 2022: **Figure 2G**), and stable clavicular/RPLNs (June 2023: **Figure 2H, I**).

### August 2024 (PD)

In July 2023, she autonomously reduced megestrol acetate to 80 mg/d and letrozole (2.5 mg Qodpo). August 2024 CT showed progressive RPLNs (2.9×2.8 cm) with stable clavicular nodes (**Figure 2J, K**).

### August 2024–until now (SD PFS3 = 12 months)

Full-dose therapy was restarted, and November 2024 CT showed reduced RPLNs (2.6×2.3 cm; **Figure 2L**). As of the last follow-up, she maintained SD with normal laboratory tests, good quality of life, and no significant AEs; OS was 108 months.

## Discussion

This report describes sustained clinical benefit exceeding 99 months of progression-free survival (PFS: PFS1 = 38 months, PFS2 = 49 months, PFS3 = 12 months) and over 108 months of overall survival (OS) in a patient with hormone receptor (HR)-positive (estrogen receptor [ER], 90%; progesterone receptor [PR], 90%) metastatic endometrioid carcinoma (EC) treated with megestrol acetate plus letrozole—with no acquired resistance mechanisms identified during treatment. To our knowledge, this represents the first case report documenting long-term sustained disease control with this novel therapeutic combination in EC.

Given that estrogen-driven signaling parallels mechanisms in hormone receptor–positive breast cancer(BC), endocrine therapy is a rational strategy for advanced HR-positive EC. The National Comprehensive Cancer Network (NCCN) guidelines recommend tamoxifen plus progestin or everolimus plus letrozole as first-line systemic therapies, with reported median overall survival (OS) of 31 months and 17 months, respectively (5–7). Progression-free survival (PFS) is notably longer in chemotherapy-naïve patients. Notably, our patient attained an OS of 108 months with ongoing disease control, substantially exceeding these benchmarks and representing the most durable endocrine therapy–mediated response reported in this setting to date. We hypothesize that three potential factors may underlie the long-term survival benefit observed in this patient.

Firstly, the combination of megestrol acetate and letrozole exerts synergistic antitumor effects in HR-high EC. Letrozole—an aromatase inhibitor—targets aromatase (upregulated in EC stroma vs normal endometrium to boost estrogen-driven growth (8, 9) to reduce estrogen production, thereby inhibiting EC cell proliferation and inducing apoptosis (10–12). Furthermore, megestrol acetate acts via progesterone receptors (PR) to regulate genes governing proliferation and apoptosis (13), with the pair dually blocking estrogen-dependent growth and PR-mediated differentiation.

In our case, disease progression (new RPLNs metastases/enlarged cervical lesions) occurred following two patient-initiated events: medication discontinuation and dose halving; reinitiating full-dose therapy restored disease stabilization—directly supporting the regimen’s efficacy. Additionally, the initial reduction in drug dosage may have impacted the therapeutic efficacy.

Furthermore, in the phase II PIONEER trial, Rebecca Burrell et al. firstly reported that megestrol plus letrozole combination therapy achieved a significantly greater reduction in Ki-67 expression versus letrozole monotherapy (P = 0.013) in HR-positive metastatic breast cancer, indicating synergistic antiproliferative activity (14). High ER/PR expression correlates with enhanced endocrine therapy efficacy (15), This patient’s 90% PR expression likely potentiated the antitumor effect of the megestrol-letrozole combination, resulting in sustained disease control. Collectively, these mechanistic insights and clinical evidence substantiate that the novel combination of megestrol acetate and letrozole exerts potent antitumor efficacy, particularly in endometrial cancer cells with high hormone receptor (HR) expression.

Secondly, the patient’s 7-month self-discontinuation of megestrol acetate-letrozole (Jan–Jul 2020, COVID-19 pandemic) may have extended treatment sensitivity via a ‘drug holiday (16). Such interruptions prolong efficacy and delay resistance—evidenced in chronic myeloid leukemia (17) and non−small cell lung cancer (18) treated with tyrosine kinase inhibitors (TKIs), consistent with drug resistance evolution principles.

To date, no studies document drug holiday improving survival in EC, but some breast cancer data may support feasibility: the phase III SOLE trial (19) showed postmenopausal postoperative HR-positive patients (7-year follow-up) had comparable DFS (Disease-Free Survival)/OS with letrozole (4 years + 3-month holiday then resumption) vs continuous dosing; with fewer distant metastases in the intermittent cohort; a Japanese study showed palbociclib dose reduction/withdrawal had no harmful effect on endocrine therapy for HR-positive advanced BC (20); and a phase II trial (NCT05305924) is investigating a 1-month drug holiday during endocrine therapy for ER-positive metastatic BC (21). Although we hypothesize that drug holidays may prolong patients’ drug sensitivity, intermittent treatment interruptions and dose reductions during the therapeutic course also precipitate rapid disease progression. Thus, for clinical management, defining suitable patient subgroups for drug holidays, optimal pretreatment duration before holidays, monitoring strategies, and appropriate holiday length require in-depth deliberation and more clinical evidence.

Finally, the patient’s minimal chemotherapy exposure (only 2 cycles due to severe toxicity) may have contributed to the benefit. This aligns with phase II data: chemo-naive EC patients on everolimus-letrozole had longer median PFS (28 vs 4 months; P<0.00179) (5), supporting the notion that prior avoidance of chemotherapy preserves endocrine sensitivity.

This case has limitations: First, megestrol acetate-letrozole efficacy in endometrial carcinoma (EC) requires validation via larger prospective trials. Second, insufficient tissue specimens precluded comprehensive molecular subtyping during the progression of the patient’s disease, preventing assessment of POLE mutation (linked to favorable EC outcomes), microsatellite instability (MSI) status, and TP53 mutation (22).Finally, the NCCN (National Comprehensive Cancer Network Guidelines) guidelines of uterine neoplasms recommend that antiestrogen therapy combined with CDK4/6 inhibitors can be used in patients with ER-positive endometrial carcinoma, given its potential for durable responses (23, 24). Meanwhile, studies have demonstrated that CDK4/6 inhibitors may overcome resistance to single-agent hormone therapy (25). Based on this, whether this combined therapeutic strategy(antiestrogen therapy combined with CDK4/6 inhibitors) can provide a novel treatment option for our case after developing drug resistance remains a subject worthy of further investigation.

Despite these constraints, our findings highlight the megestrol-letrozole combination as a potentially superior endocrine strategy for recurrent metastatic hormone receptor (HR)-positive EC, achieving unprecedented long-term disease control (sustained remission > 9 years) for the first time. High ER/PR expression (ER 90%, PR 90%), holiday drug, and chemotherapy-naïve status may collectively identify a patient subgroup with enhanced therapeutic responsiveness. Prospective trials are needed to validate these findings and refine patient selection.

## Data Availability

The original contributions presented in the study are included in the article/supplementary material. Further inquiries can be directed to the corresponding author.
